# Declining drinking among adolescents: Are we seeing a denormalisation of drinking and a normalisation of non-drinking?

**DOI:** 10.1111/add.15611

**Published:** 2021-07-14

**Authors:** Gabriel Caluzzi, Michael Livingston, John Holmes, Sarah MacLean, Dan Lubman, Paul Dietze, Rakhi Vashishtha, Rachel Herring, Amy Pennay

**Affiliations:** 1 Centre for Alcohol Policy Research, La Trobe University, Bundoora, Victoria, Australia; 2 National Drug Research Institute, Curtin University, Perth, Western Australia, Australia; 3 School of Health and Related Research, University of Sheffield, Sheffield, UK; 4 School of Allied Health, Human Services and Sport, La Trobe University, Bundoora, Victoria, Australia; 5 Turning Point, Eastern Health, Melbourne, Victoria, Australia; 6 Monash Addiction Research Centre, Eastern Health Clinical School, Monash University, Melbourne, Australia; 7 Behaviours and Health Risks Program, Burnet Institute, Melbourne, Victoria, Australia; 8 Drug and Alcohol Research Centre, Middlesex University, London, UK

**Keywords:** Adolescent, alcohol, declining drinking, denormalisation, non-drinking, normalisation

## Abstract

**Background:**

In the early 2000s, alcohol use among young people began to decline in many western countries, especially among adolescents (ages between 12–17 years old). These declines have continued steadily over the past two decades, against the backdrop of much smaller declines among the general population.

**Argument:**

Hypotheses examining individual factors fail adequately to provide the necessary ‘big picture’ thinking needed to understand declines in adolescent drinking. We use the normalisation thesis to argue that there is strong international evidence for both processes of denormalisation of drinking and normalisation of non-drinking occurring for adolescents in many western countries.

**Conclusions:**

Research on declining adolescent drinking provides evidence of both denormalisation of alcohol consumption and normalisation of non-drinking. This has implications for enabling policy environments more amenable to regulation and increasing the acceptability of non-drinking in social contexts. Normalisation theory (and its various interpretations) provides a useful multi-dimensional tool for understanding declines in adolescent drinking.

## Introduction

In the early 2000s, alcohol use among young people began to decline in many western countries, especially among adolescents (ages 12–17 years old). These declines have continued steadily over the past two decades [1–4] against the backdrop of much smaller or non-existent declines among the general population, stimulating numerous hypotheses examining changes influencing adolescent drinking [5–8]. Despite being important mechanisms that may have contributed to the decline, many of these factors alone (e.g. changes in parenting or digital technology) fail to provide the necessary ‘big picture’ thinking needed to understand these declines [9,10]. Just as earlier research used the concept of normalisation [11,12] to elucidate regular and heavy drinking styles among young people [12–14], recent empirical work has applied the concepts of denormalisation of drinking [15] and normalisation of non-drinking [16] to better understand declines in adolescent drinking. In this debate piece, we argue that there is strong international evidence for two processes—denormalisation of drinking, and normalisation of non-drinking—occurring for adolescents in many western countries since the turn of the millennium.

### Alcohol use and the markers of (de)normalisation

In their original work, Parker *et al*. [12] drew on longitudinal data with young people in Northwest England in the 1990s. They proposed a normalisation thesis to describe changes in the use of, and attitudes toward, recreational drug use. Normalisation suggested that what had previously been considered ‘deviant’ or subcultural practices—such as heavy drinking and recreational illicit drug use—had become more acceptable in mainstream youth culture. This meant that more young people had access to, were willing to try, and used these substances to a greater extent [12].

Parker and colleagues’ normalisation thesis identified connections across a range of variables concerning substance use and attitudes toward it, including broad social and economic changes to young people’s lives [17]. In the past 20 years, normalisation has been used as a framework for understanding changing patterns of use and attitudes toward substances (both licit and illicit) across a range of contexts [18,19]. Although young people’s reasons for drug and alcohol use are complex, shaped by social, cultural and economic conditions [20–22], normalisation theory can be seen as a ‘barometer of change’ [11] to highlight how substances, styles of use and social acceptability change in different countries over time [23]. Therefore, we suggest normalisation theory can bring together and contextualise various factors and mechanisms found in empirical research to elucidate declining alcohol use among adolescents.

Based on their thesis, Parker and colleagues created the following criteria as a way of assessing processes of normalisation:

1. increased experimentation or ‘trying’ rates,
2. high levels of recent or regular use,
3. high availability and accessibility,
4. high levels of social accommodation (i.e. liberal attitudes toward the substance among young people, particularly non-users),
5. high levels of cultural accommodation (i.e. positive [or at least neutral] portrayals of the substance in mainstream media, and accommodating attitudes in the general population) and
6. more relaxed policy or enforcement (*we have combined this tenet with 3—availability and accessibility—given their close connection in the alcohol space).

The first three normalisation criteria focus on use and access to a substance, whereas the latter three point toward broader societal acceptance of substance use. This is important because changes in substance use and societal acceptance of substance use often happen independently or in opposite directions without normalisation occurring (i.e. without all of Parker et *al.’s* tenets having been met). For example, in Australia, the ‘personal approval of regular cannabis use’ has increased steadily since 2007; however, at the same time, cannabis use has almost halved among 14- to 19-year-olds [1,24]. Similarly, in the United Kingdom (UK), the general community were found to exhibit more tolerant attitudes toward cannabis during the 2000s, while in 2009 it was rescheduled from a Class C to a Class B drug (a more dangerous class) [25]. In the opposite direction, heroin use has been rising in the United States (US) since 2007 [26] without a corresponding rise in its social or cultural accommodation. These findings broadly indicate that normalisation of cannabis and heroin have not occurred as not all criteria have been met.

We propose two ways of applying normalisation theory to adolescent drinking. The first is denormalisation of drinking, where drinking becomes a less accepted, even atypical practice [27]. This would imply decreased experimentation and use of alcohol, efforts (both policy and otherwise) to make alcohol less available and accessible, and less accommodating social and cultural attitudes toward adolescent drinking. The second is normalisation of non-drinking, wherein abstinence comes to be seen as a normal and accepted practice. This would imply increase rates of abstention alongside greater social and cultural acceptance of non-drinking. The latter process is different to the denormalisation of drinking, because it indicates that the acceptance of abstinence could occur alongside a continued acceptance of drinking. Therefore, these can be considered related but separate processes; denormalisation of drinking may enable more normalised non-drinking cultures and vice versa. However, non-drinking may also become normalised in a climate that does not necessarily denormalise drinking. We draw on recent empirical work (both quantitative and qualitative) to assess the evidence for each.

### Experimentation with alcohol among adolescents

The concept of ‘experimentation’ as it applies to illicit drug use (i.e. ‘trying’ something new) is difficult to apply to alcohol use as the different ways adolescents taste, sip, or drink alcohol are rarely distinguished [28]. However, data on initiation of alcohol use measured by having had a full serve is a useful proxy for experimentation rates. Recent studies from northern European and English-speaking countries have shown that adolescents are delaying initiation of alcohol use. The proportion of those who have started drinking before the age of 13 (early onset) has decreased in most European countries during the past 20 years [2]. In the United States, the average age at first drink increased by 6 months between 2000 and 2015 [29]. Australian data show even larger changes, with the age of initiation increasing from 14.9 in 2001 to 16.4 in 2016 [30], whereas young UK drinkers’ age at first drink increased by ~1 year in the same period [31].

An increasing proportion of adolescents also report not having commenced alcohol use. Lifetime abstention (i.e. never having had a drink) for 14- to 19-year-olds in Australia increased from 22% to 49% between 2001 and 2019 [1], with similar trends evident in the United Kingdom [32] and Europe [2]. For the 30 countries that have consistently completed the European School Survey Project on Alcohol and Other Drugs (ESPAD) between 2003 and 2019, overall lifetime abstention among 15-year-olds more than doubled in this time [2].

Generally, younger age at first drink and early experiences of intoxication have been closely linked to heavier use as adolescents grow older [33,34]. However, evidence is mixed as to whether recent declines in adolescent experimentation with alcohol have led to abstention or reduced drinking in adulthood [35–37]. This paints a complex picture; adolescents may be less likely to initiate drinking during adolescence, but open to using alcohol as they grow older. Some qualitative research supports this, with young abstainers highlighting disapproval for drinking in people under 18 (the legal age in most countries) as a motivator for non-drinking [10,16,38]. Although adolescents may drink as they get older, delays in experimentation with alcohol appear to represent a process of denormalisation of drinking for younger adolescents.

### Recent or regular use of alcohol among adolescents

As well as declines in experimentation, many western countries have recently seen declines in the frequency of adolescent drinking. For example, the prevalence of weekly alcohol use for 15 to 16-year-olds fell significantly between 1999 and 2015 in four of five regions in Europe, with declines especially sharp after 2007 [39]. Monthly drinking for adolescents also declined in Australia between 2002 and 2017 from 49% to 27%, in New Zealand between 2002 and 2012 from 55% to 31%, in Canada between 2002 and 2014 from 45% to 28%, and in the United States between 1999 and 2017 from 50% to 30% [40]. The cross-national Health Behaviours in School-aged Children (HBSC) survey spanning 36 countries also noted a large reduction in past week drinking for 15-year-olds in most countries and regions between 2002 and 2014 [3].

Heavy drinking measures show similar patterns. For example, in the United States, past-month ‘binge drinking’ (five or more drinks on an occasion) among 12- to 17-year-olds fell by more than half from 26% in 2002 to 12% in 2016 [41]. The HBSC survey shows declines in self-reported experience of lifetime drunkenness (two or more occasions) for 15-year-olds in 28 of 32 countries that provided data in 2002 and 2018, including steep drops in northern and eastern Europe where rates fell by more than half in many countries [42,43].

In their work on adolescent drinking and cigarette use, Sznitman *et al*. [15] suggested that substance use prevalence rates of 40% or above at a population level implies a meaningful threshold for substance use normalisation, because at this point the social and demographic factors between users and non-users become less distinguishable [44]. Recent, regular, ‘binge’ drinking, and experience of drunkenness, have fallen below this threshold for adolescents in many countries. For example, recent drinking among 12- to 17-year-olds in Australia, New Zealand, Canada and the United States dropped below 40% between 1999 and 2017 [40]. For 15-year-olds in the most recent HBSC report (2018) across 45 jurisdictions, only boys from Denmark reported a prevalence of over 40% of at least two occasions of drunkenness in their lives, and no countries reached this measure for girls [43].

There remains substantial variation between countries in the scope and magnitude of reported declines. Declines seem particularly sharp in northern European and English-speaking countries, with trends elsewhere less stark [40]. Although we should not treat consumption trends as monolithic, epidemiological data provide a compelling picture of declines in regular use of alcohol in the past 15 to 20 years, to levels well below those previously cited as evidence of ‘normalisation’. Therefore, survey data evidence would suggest that the reverse process is occurring for adolescents (i.e. denormalisation of drinking).

### Availability and accessibility of alcohol, and policy and enforcement

Young people have long been the focus of alcohol policies, both in terms of specific interventions (e.g. minimum legal drinking age) and via broader policies like price interventions [14,45,46]. Studies have generally shown increasing public support for restrictive alcohol policies focused on young people during the period of the decline in adolescent drinking [47–50], even among young people themselves [51–53].

Although there has been no attempt to systematically assess policy changes internationally, there are clear examples of policies in particular countries aiming to reduce the availability of alcohol to adolescents. These include raising the purchase age, secondary supply or social host liability laws, taxing of youth-oriented drinks, underage possession laws and strict enforcement of proof-of-age identification checks at point of sale [54–61]. Although secondary supply laws have not shown evidence of driving reductions in adolescent drinking in Australia [62], they may have had an indirect effect with recent studies identifying a significant reduction in parental supply of alcohol to adolescents over the past 15 years [63,64]. Perhaps as a result of stricter regulation around supply, the decline in adolescent drinking has also reduced perceived availability of alcohol in some countries [65,66]. Although many adolescents still perceive alcohol to be readily available [2], parental supply of alcohol has decreased in some countries [64,67,68] and in others there are perceptions of reduced supply of alcohol among social networks (such as through friends and siblings) [66].

National health advice on adolescents’ drinking has also become more restrictive in some countries. In 2001, Australia’s National Health and Medical Research Council Guidelines stated that young people under the age of 18 should not exceed low-risk drinking guidelines [69]. The revised 2009 guidelines suggested that ‘for young people ages 15 to 17 years, the safest option is to delay the initiation of drinking for as long as possible’ [70]. In the recently released 2020 draft guidelines, this changed to ‘young people under 18 years of age should not drink alcohol’ [71]. The UK guidelines similarly became more restrictive when they were updated in 2009 [72]. The movement toward more stringent policy guidelines, increased regulation, and stricter policies for adolescent drinking indicates a denormalisation of underage drinking both in terms of reduced availability and accessibility, and in terms of stricter policy and enforcement.

### Social accommodation

Social accommodation is a measure of the acceptability of drinking, and for our purposes here, non-drinking, by others. A recent New Zealand study using nationally representative health survey data found that the decreasing acceptance of alcohol use by adolescents was the most important contributor to declines in heavy adolescent drinking [73]. A recent Swedish study showed decreasing drinking among peers accounted for reduced drinking among adolescents, highlighting the importance of non-drinking social networks [67]. Recent qualitative research has suggested that younger adolescents (more so than older adolescents) are increasingly perceiving drinking as uncommon, risky and deviant [16,38], suggesting social accommodation of alcohol is sensitive to age.

There is evidence for decreasing approval from parents toward adolescent drinking. Changes in parental attitudes toward alcohol, including reduced supply of alcohol to children, increased monitoring and setting alcohol norms have been linked to declines in adolescent drinking in quantitative studies [74,75]. As indicated earlier, some countries have observed a significant decrease in parental supply of alcohol and increase in parental restrictiveness toward adolescent drinking [64,65,76]. Qualitative research reports that parents are increasingly incorporating dialogue, monitoring and dissuasion of heavy drinking as strategies to reduce drinking [10,16,65,77,78].

Past research has highlighted some of the difficulties young abstainers face in social situations, leading to them avoiding drinking environments, providing excuses for non-drinking, or ‘passing’ as drinkers [79–83]. In doing this, young non-drinkers reproduced drinking as normative and mainstream [84]. However, recent studies focusing on young light and non-drinkers suggest there are a growing number of ways to socialise without alcohol and fewer social sanctions for not drinking [10,85–87]. Many adolescents in these studies supported their non-drinking with positive notions of choice and individuality, highlighting how non-drinkers may be redefining what is considered ‘normal’ [84,88]. Adolescents may be finding new avenues to challenge previous social norms around drinking and intoxication, signalling a growing normalisation of non-drinking in social contexts.

### Cultural accommodation

The alcohol industry’s extensive marketing of alcohol in western countries has been linked to the cultural normalisation of adolescent drinking in the 1990s and early 2000s [89]. However, increased visibility of public health experts and advocates in mainstream media since the mid-2000s has led to greater problematisation of drinking in Australian and UK media [90–92]—although the alcohol industry’s efforts to normalise alcohol are ever-present [93]. The alcohol industry has also responded to health movements by promoting alcohol-free and low alcohol-content drinks [94]. Although there is little evidence about how widely alcohol-free drinks are used among young people, they may represent a symbolic accommodation of non-drinking.

Greater attention to health and alcohol in prevention programs, policy and media depictions may have also made adolescents increasingly ‘drugwise’ of the harmful effects of alcohol [7]. Although there is limited evidence that adolescents have become more health conscious [8], the health and societal effects of alcohol have become increasingly important for young people [16,86,95,96] and ESPAD data has shown European adolescents perceive the risks related to alcohol use as greater than in the early 2000s [2]. Recent qualitative research shows that concerns about physical, social and mental health are considered legitimate reasons for not drinking [96–98]. Changing societal norms around drinking and driving have also been linked with reduced consumption [99], with adolescents drawing on drunk-driving as a reason to avoid alcohol [97].

Online platforms have also become an integrated part of adolescent cultures in ways that both normalise and challenge alcohol use. Earlier research showed how social media encouraged displays of drinking [100] and exposed adolescents to alcohol advertising [101]. More recent research suggests that social media also brings with it a range of complexities around self-presentation, future employment, censorship and engagement with risk that discourages drinking and displays of drunkenness [86,102]. Greater alcohol use among heavy social media users suggests social media platforms continue to reinforce pro-alcohol norms and encourage socialising with alcohol [103]. Alternatively, gaming and other technology-based leisure options may also provide a normalised way of doing leisure without drinking [9,103].

Changing social roles may further influence the cultural accommodation of alcohol. Research suggests young men today are less reliant on alcohol for performing masculinity [96,97,104]. Although recent feminist discourses may have challenged gender stereotypes around alcohol use [105], in some countries and contexts women’s drinking is still morally problematised [106,107]. Because the cultural mechanisms that reduce drinking are likely to be different for young men and women [10], it may be that non-drinking among young men has become more accepted and normalised, whereas young women’s drinking continues to be problematised and denormalised.

Young people are also required to adapt to broad changes in the education and labour markets, and there is some suggestion that this may have devalued alcohol [108,109]. Recent qualitative research shows young non-drinkers emphasise self-control as a way to manage health, image and social interactions [16,85,87,98,102]. This runs contrary to theorisations that suggest drinking is a way for young people to escape social pressures and resist governing forces [110,111]. Indeed, contemporary adolescents may be navigating a performance culture that is less accommodating of moments of hedonism through drinking and intoxication, and therefore, normalises non-drinking.

## Conclusions

Normalisation theory shows how various empirically identified factors and mechanisms of change co-exist and co-produce to provide an overarching theory that more holistically accounts for declines in adolescent drinking. We suggest that research on declining adolescent drinking provides evidence for both denormalisation of drinking and normalisation of non-drinking across Parker *et al*.’s key criteria. On the one hand, adolescents are drinking less and pursuing intoxication less frequently, and alcohol seems to be a less central feature of their lives. On the other hand, more young people are choosing to abstain and are experiencing less stigma as a result of their abstinence, even in contexts where alcohol use might have previously been perceived as normal (such as parties). This highlights the interrelated, yet distinct nature of these two processes. As we show in Table 1, denormalisation seems to be more supported by epidemiological data, whereas evidence for normalisation of non-drinking can be found in qualitative studies suggesting increasingly positive social and cultural environments for abstainers. The concept of ‘differentiated normalisation’ [21] usefully captures how declines have varied in manifestations and across subgroups. For example, it seems that regular or heavy drinking is subject to more apparent processes of denormalisation than ‘any’ use [73,112,113]. There also seems to be evidence of age-specific denormalisation in policy efforts to reduce underage drinking and increased perceptions of alcohol as a harmful substance among younger adolescents [16,38,73,95].

From a public health perspective, the denormalisation of alcohol use, particularly in the context of increased health concerns (like cigarette smoking), may have created an environment more open to regulation [114]. This might have enabled greater support for reinforcing alcohol policies that specifically target younger people. Moreover, the age-specific denormalisation we highlighted may have implications for how alcohol policies and interventions target certain age groups (e.g. focusing on young adults as well as younger adolescents). Alternatively, public health efforts that encourage adolescents to enjoy social contexts without alcohol and destigmatise non-drinking may create a more positive environment for non-drinkers [85]. Normalising non-drinking (rather than denormalising drinking) may also support a more heterogeneous drinking culture that both reduces alcohol-related harms and avoids marginalising abstainers or heavier drinkers [115].

It is important to note that, had we explored literature on regular or heavy drinkers, we may have uncovered little change, or even increases, in drinking among some samples. For example, there is some evidence that declines in adolescent drinking have occurred at proportionately different rates among heavier and lighter drinkers [116–118]. The macro focus of normalisation may overlook increasingly fragmented drinking cultures where pockets of micro normalisation continue, suggesting a continuing need in policy to focus on structural interventions [119]. We also note that our analysis is specific to high income, largely western countries. Elsewhere, trends and associated meanings of alcohol would likely reflect significant social and cultural differences.

Normalisation was relevant to the United Kingdom in the 1990s because change was fast, clear and one-directional [25]. Similarly, declining trends in adolescent drinking have been apparent across numerous countries in a comparable time frame. These international parallel trends call for theory that can account for a range of interrelated macro and micro-level changes—something that we have not yet seen in the literature. By drawing on a range of quantitative and qualitative studies, we suggest Parker and colleagues’ normalisation thesis (and its variations) is a useful few multi-dimensional theory for understanding these declines, with strong evidence for both processes of normalisation of non-drinking and denormalisation of drinking among adolescents.

## Figures and Tables

**Table 1:** Evidence for different forms of normalisation.

Criteria	Evidence for denormalisation of alcohol	Evidence for normalisation of nondrinking	Evidence for differentiated normalisation
Experimentation with alcohol among adolescents	Delayed initiation or experimentation Increases in abstention Policy and parenting attempts to delay drinking initiation	Increases in abstention	Delay in initiation not always persisting into adulthood
Recent or regular use of alcohol among adolescents	Reduced prevalence in recent, regular and heavy use		Trends starkest in northern European and English-speaking countries
Availability and accessibility of alcohol, and policy and enforcement	Support for restrictive alcohol policies Reduced parental supply Stricter regulation and enforcement of policies targeting adolescent drinking		
Social accommodation	Increase in negative attitudes toward adolescent drinking Less socially accommodated by parents	More ways to socialise without alcohol Less social sanctions around non-drinking	Social accommodation of alcohol sensitive to age
Cultural accommodation	Increased health and social problematisation of alcohol	Accommodation of non-drinkers by alcohol industry Increasing legitimisation of health as reason for non-drinking New ways to perform masculinity without alcohol Non-drinking aligning with performance culture	
